# Development and evaluation of a soft pneumatic muscle for elbow joint rehabilitation

**DOI:** 10.3389/fbioe.2024.1401686

**Published:** 2024-10-24

**Authors:** Mostafa Orban, Kai Guo, Caijun Luo, Hongbo Yang, Karim Badr, Mahmoud Elsamanty

**Affiliations:** ^1^ School of Biomedical Engineering (Suzhou), Division of Life Sciences and Medicine, University of Science and Technology of China, Hefei, China; ^2^ Suzhou Institute of Biomedical Engineering and Technology, Chinese Academy of Sciences, Suzhou, China; ^3^ Mechanical Department, Faculty of Engineering at Shoubra, Benha University, Cairo, Egypt; ^4^ Mechatronics Engineering Department at Modern University for Technology and Information-MTI, Cairo, Egypt; ^5^ Mechatronics and Robotics Department, School of Innovative Design Engineering, Egypt-Japan University of Science and Technology, Alexandria, Egypt

**Keywords:** soft robotics, pneumatic actuators, elbow rehabilitation, biomechanical simulation, finite element analysis (FEA), deep learning, motion tracking, kinematic analysis

## Abstract

Elbow joint rehabilitation presents a formidable challenge, underscored by the joint’s complex biomechanics and high vulnerability to injuries and degenerative conditions. Despite the advancements in rehabilitative technology, current solutions such as rigid exoskeletons often fall short in providing the precision, flexibility, and customization needed for effective treatment. Although traditional robotic aids, such as rigid exoskeletons, help recover, they lack in providing sufficient flexibility, comfort, and easy customization with no need for complicated calculation and complex design considerations. The introduction of soft pneumatic muscles marks a significant development in the rehabilitation technologies field, offering distinct advantages and unique challenges when compared to conventional rigid systems. These flexible actuators closely mimic the elasticity of biological tissues, improving safety and interaction between humans and machines. Designed for individualized therapy, its versatility allows application in various rehabilitation scenarios, from clinical settings to home settings. The novelty of this approach lies in the development of biomechanically-compliant soft pneumatic muscles optimized for precise rotational control of the elbow joint, coupled with an advanced deep learning-based motion tracking system. This design overcomes limitations in force control, stability, and pressure requirements found in existing pneumatic-based systems, improving the safety and efficacy of elbow rehabilitation. In this study, the design, fabrication and systematic evaluation of a soft pneumatic muscle for elbow rehabilitation are presented. The device is designed to closely simulate the complex biomechanical movements of the elbow, with a primary focus on the rotational motions that are essential for controlling flexion and extension, as well as positioning the wrist during grasping tasks. Through the integration of precise geometric parameters, the actuator is capable of controlled flexion and extension, reflecting the natural kinematics of the elbow. Employing a rigorous methodology, the research integrates finite element analysis with empirical testing to refine the actuator’s performance. Under varying air pressures, the soft muscle demonstrated remarkable deformation along the X-axis (10–150 mm) and the Y-axis, indicative of its symmetrical rotational behavior, while maintaining minimal elongation along the Z-axis (0.003 mm max), and proper lifiting force under a maximum wight of 470 gm. highlighting the stability and targeted response of the device to pneumatic actuation. A specialized experimental apparatus comprising a 3D environment, a pneumatic circuit, a LabVIEW-based control system, and a deep learning algorithm was developed for accurate position estimation. The algorithm achieved a high predictive accuracy of 99.8% in spatial coordination tracking, indicating the precision of the system in monitoring and controlling the actuator’s motion.

## 1 Introduction

**FIGURE 1:**
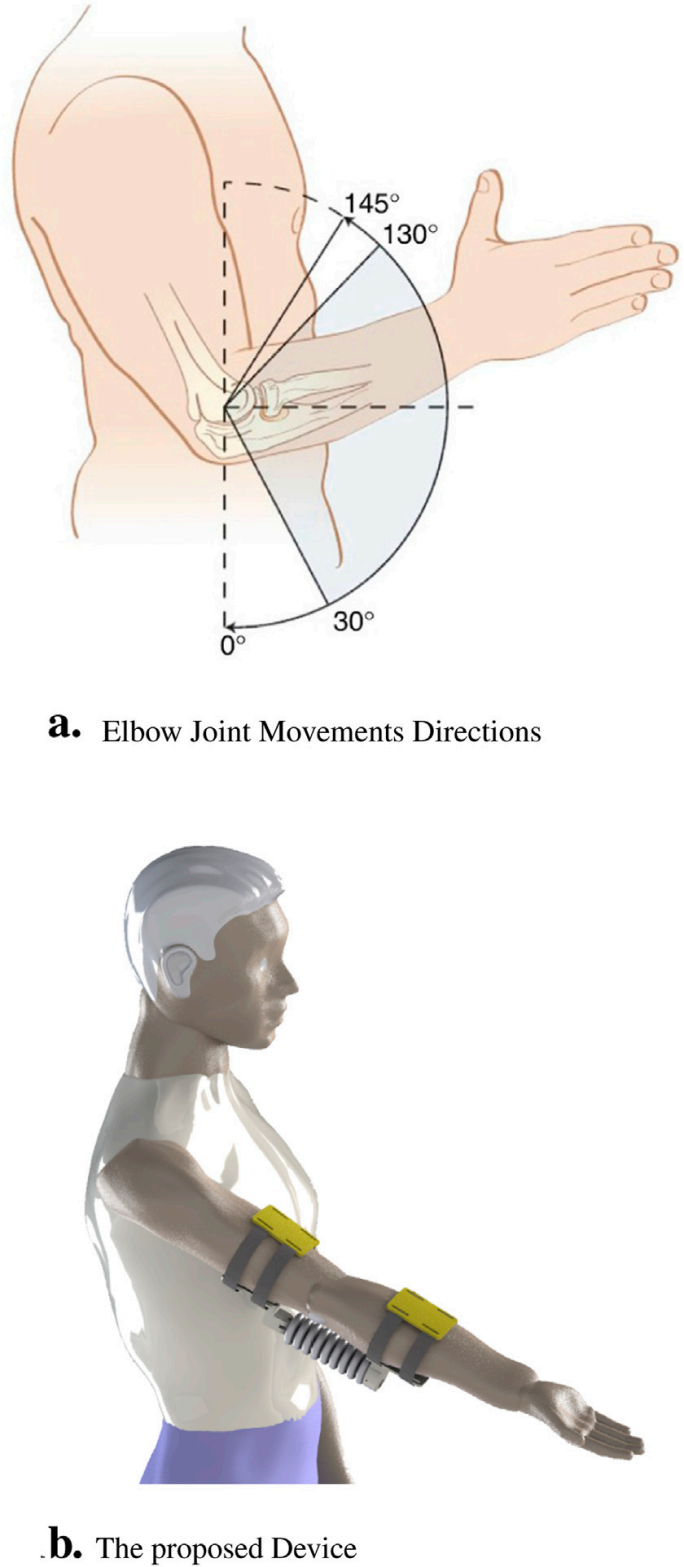
The real application for our designed muscle explaining the following components. **(A)** Illustration that highlights the different angles of movement in the elbow joint, indicating rotational flexion and extension. **(B)** Representation of the device designed specifically for elbow muscle control and movement.

The elbow joint, defined as a trochoginglymoid and diarthrotic hinge joint, engages in a complex interaction between the skeletal, musculotendinous, capsuloligamentous and neurovascular constituents. These anatomical elements, as shown in Figure 1, underlie the distinctive biomechanical functions and pathological manifestations associated with elbow disorders. These disorders include degenerative conditions, cumulative stress injuries, such as tendinopathies, and traumatic events (Bowers et al., 2023). In this context, our study aims to improve the precision of clinical diagnoses and the efficacy of conservative treatment approaches for these diseases. The biomechanics of the elbow are based on two principal actions: flexion and extension. These motions are orchestrated by the concerted action of the brachii biceps, brachioradialis, and brachialis, which facilitate elbow bending. At the same time, the triceps muscle allows for extension or straightening of the arm. Pathological conditions, including lateral epicondylitis (Tennis elbow), medial epicondylitis (Golfer elbow), fractures, dislocations, Cubital Tunnel Syndrome, cerebrovascular incidents, and osseous impairments, can compromise the functional capacity of the elbow, leading to a decrease in range of motion and dexterity. These deficits can manifest as pain, weakness, restricted mobility, and difficulty in the performance of daily activities, which require comprehensive rehabilitation protocols to restore patient functionality.

Rehabilitation of the elbow joint, particularly in cases of injury, degeneration, or limited mobility, presents a significant challenge.Rehabilitating the elbow joint represents a significant challenge due to its complex anatomy and susceptibility to a range of injuries and degenerative diseases. Statistical studies announced that the global incidence of elbow dislocations in the normal population is approximately 6/100,000; nearly half of these cases are related to sports. 26% of elbow dislocations have associated fractures, The elbow is ranked fifth among the most common body parts injured in young person’s reflecting a considerable public health concern (Almalki et al., 2020). The demographic most affected includes adolescents aged 10 to 19, who account for nearly 44.5% of these cases, predominantly during sports activities. The limited mobility associated with elbow impairments where a restricted range of motion from 75 ° to 120 ° allows only 49% of affected individuals to perform essential daily tasks underscores the profound impact on functional ability and quality of life. Current rehabilitation protocols often fall short in terms of precision, adaptability, and the ability to restore full range of motion, which are critical for effective recovery. These limitations highlight an urgent need for innovative rehabilitation solutions that can offer improved adaptability, durability, and structural integrity to fully restore elbow function (Stoneback et al., 2012; Vasen et al., 1995; Ponkilainen et al., 2022; Guo et al., 2022; Masengo et al., 2020). Traditional rehabilitation methods often incorporate robotic assistance, mainly using rigid exoskeletons. Although these methods are beneficial to some extent, they have limitations in terms of flexibility, comfort, and patient-specific customization (Martinez-Hernandez et al., 2021). Recognizing these limitations has led researchers to explore alternative technologies that resulted in the emergence of soft pneumatic muscles. Unlike rigid systems, these novel mechanisms offer flexibility and versatility, adequately meeting the unique demands of elbow rehabilitation. Their ability to bend, twist, and stretch makes them adaptable and suitable for confined spaces. The use of flexible materials that mimic biological properties further enhances safety and adaptability in human interactions. As such, these systems are suitable for a variety of rehabilitation settings, from clinical settings to outdoor settings (Orban et al., 2023).

Rigid elbow exoskeletons have been extensively studied in both rehabilitation and assistive devices due to their ability to provide highly controlled and stable movements. One of the key advantages of rigid exoskeletons is their capacity to generate significant torque and handle heavy loads, making them suitable for applications requiring force amplification or precise control over joint trajectories (Orban et al., 2020). Examples include systems such as the ARMin exoskeleton (Nef et al., 2007), which has been shown to improve motor recovery through repetitive guided arm movements in stroke patients, and the EXO-UL8 (Chiou et al., 2023), which provides upper-limb support with an emphasis on strength augmentation for rehabilitation and assistive purposes. However, despite these advantages, rigid exoskeletons face several limitations. Notably, their lack of compliance makes them less adaptable to the user’s natural joint movements, raising concerns about safety and comfort, particularly during prolonged use (Lobo-Prat et al., 2014). The rigid structure can restrict degrees of freedom, resulting in less naturalistic movement patterns, which may not be ideal for tasks requiring fine motor control or intricate motion (Veneman et al., 2007). Additionally, the weight and bulkiness of rigid systems often lead to user fatigue, reducing the suitability of these devices for long-term wear or mobile applications (Dollar and Herr, 2008). In contrast, soft exoskeletons offer enhanced wearability, comfort, and adaptability, making them more feasible for daily activities and continuous rehabilitation. These trade-offs highlight the need for innovations that balance the precision and force output of rigid systems with the flexibility and comfort of soft exoskeletons, particularly for rehabilitation and assistive applications.

One of the key advantages of these soft robotic systems lies in their design process. The absence of rigid components and intricate links significantly improves safety in human-robot interactions (Hashem et al., 2021; Elsamanty et al., 2023). Coupled with their precision and controlled assistance, soft robotics demonstrate promising potential in the field of medical assistive devices and upper limb rehabilitation (Zhang et al., 2023). The design of these soft robots often draws inspiration from biological organisms with the aim of replicating and enhancing their movements. This approach contributes significantly to research and supports technological advancements in the field of rehabilitation (Drotman et al., 2019). However, it is important to recognize that the deformation behavior of these soft robots is determined by a complex interplay of several factors, including material selection, fabrication techniques, and interior chamber design. Each of these factors plays a vital role in determining the overall performance and functionality of robots (Case et al., 2015; Elango and Faudzi, 2015; Polygerinos et al., 2015a; Manns et al., 2018; Saleh et al., 2020a; Mohamed et al., 2022). Understanding and accurately modeling these interactions is essential to advance the field of soft robotics, particularly for rehabilitation applications (Mohamed et al., 2022). By exploring and optimizing these elements, researchers aim to significantly improve the performance, versatility, and capabilities of soft robotic systems. The primary focus of this enhancement is the development of assistive soft pneumatic muscles (Polygerinos et al., 2015a; Polygerinos et al., 2015b). These advances could revolutionize the healthcare industry by enabling the creation of robots that provide safe and effective support to people throughout their rehabilitation process, improving the quality of care and patient outcomes.

Muscle actuators, designed with soft and flexible materials, play a crucial role in ensuring safe user interactions and providing variable compliance. They have found versatile applications in both robotic and medical settings (Tawk et al., 2018; Palmić and Slavič, 2023). In the field of physical therapy, these actuators have become a crucial tool to improve the rehabilitation process. They effectively alleviate fatigue and provide essential support during rehabilitation sessions for people recovering from conditions such as stroke (Guan et al., 2020). This support not only aids in motion but also manages workloads and reduces the risk of injuries, thus contributing significantly to the rehabilitation process (Guan et al., 2020; Guo et al., 2023; Orban et al., 2022).

Given these constraints, the design parameters of soft robotic muscles have become a focus of attention. Factors such as muscle length, diameter, weaving angle and choice of weaving fabric play a crucial role in determining its functionality and significantly influence the static force output (Mohamed et al., 2022; Pan et al., 2022). These factors collectively inform the performance characteristics of the muscle and its suitability for specific applications. In response to the inherent limitations of previous muscles such as McKibben muscle, recent advances in soft robotic muscle technology have facilitated the development of innovative designs that improve force output (Chen and Ushijima, 2014). These novel designs contribute to higher levels of assistance and support during rehabilitation, thus broadening the scope of applications for these muscles (Elsamanty et al., 2021). An essential aspect of these advances is the integration of pneumatic control systems and advances in materials science. This integration has mitigated the need for a compressed air tank, thereby augmenting the practicality and user-friendly nature of these soft actuators in various orthotic systems.

The versatility of soft robotic muscles extends far beyond their utility in medical settings. These muscles have found applications in a wide range of fields, including prosthetics, wearable devices, and industrial automation. Due to their adaptability, compliance, and safety advantages, they have become a suitable choice for a multitude of applications. As the field of soft robotics continues to progress, researchers and engineers are constantly exploring new design approaches, material innovations, and control strategies (Soliman et al., 2021; Youssef et al., 2022). These efforts aim to further enhance the capabilities and performance of these actuators, pushing the limits of what is achievable in this field. Consequently, there has been a paradigm shift in the design and characterization of parameters with the advent of soft muscles in the field of rehabilitation. A noteworthy study highlighted this change, where researchers examined how alterations in the air pillow angle of inclination affected both the soft muscle’s work envelope and the reaction force applied to the soft muscle’s tip. They achieved this by applying positive vacuum and vacuum pressure to the inner surfaces of the soft muscle, further highlighting the versatility of these muscles in rehabilitation settings (Saleh et al., 2020b; Tang et al., 2007).

Parallel to these developments, significant advances have been made in rehabilitation technology, such as the advent of a lightweight and cost-effective robotic system, made entirely from silicone rubber 100%. This system, specifically designed to assist in elbow movement, features a specialized actuator intended to be comfortably worn around the arm and forearm and secured with silicone bands. The primary innovation of this design is its ability to aid movements of the upper extremities, facilitating elbow flexion and extension. The use of pneumatic air control within soft components, coupled with a pressure sensor that provides real-time feedback, improves the performance of the system (Saleh et al., 2020a). Furthermore, the system is designed to precisely monitor the angles of motion through visual data captured by a webcam, thus improving the overall efficacy of the system (Oguntosin et al., 2015). Building on these advances, researchers developed an active soft ankle-foot orthotic system driven by pneumatic artificial muscles to assist with gait. This device, constructed with soft and flexible materials that mimic the structure of muscles, tendons, and ligaments, demonstrated the ability to achieve significant dorsiflexion from the resting position of the ankle joint (Park et al., 2011). This milestone achievement further highlights the transformative potential of soft robotic muscles in the field of rehabilitation.

Patients who undergo total knee arthroplasty (TKA), a surgical procedure designed to restore function to a damaged or diseased knee, often face postoperative challenges such as reduced range of motion and weakened quadriceps strength. To address this, a novel solution was developed in the form of a lightweight pneumatic exoskeleton for patients with TKA. This development presented a less cumbersome alternative to traditional metal-based exoskeletons. The exoskeleton, integrated with electronics and powered by a pneumatic fabric actuator, detects the patient’s intention of movement and provides continuous support, thus enhancing the patient’s recovery process (Ezzibdeh et al., 2019). Further advances were made with the design of a foldable pneumatic bending actuator (FPBA) using thermoplastic polyurethane fabric materials (TPU), specifically for an FPBA-based knee exosuit. Despite initial limitations in tethering due to a miniature pump, this innovative design demonstrated its ability to generate bending motions with a wide range of motion and provide human-scale torque levels at low input pressures (Fang et al., 2019).

Parallel to these developments, researchers have begun innovative initiatives in other areas of rehabilitation. A notable example is the creation of a soft elbow rehabilitation trainer that uses soft pneumatic actuators as bending joints designed specifically for elbow rehabilitation purposes. The trainer’s adaptive capabilities, combined with its self-alignment prowess, greatly expedite device adjustment processes. Furthermore, its adaptive position feedforward and feedback control mechanisms allow the elbow trainer to provide adaptive assistance and promote human-robot interaction (HRI) without requiring explicit torque and stiffness information (Wilkening et al., 2015). On the basis of the information presented above, it is evident that pneumatic rotary and foldable actuators have attracted significant interest within the research community. Their remarkable flexibility of motion, capable of operating in a wide range of bending degrees, makes them well suited for various rehabilitation and assistance applications. Although these actuators have notable advantages, such as adaptable stiffness, portability, comfort, and ultralight design, they generally operate at lower pressures, such as 0.5 bar (Thalman et al., 2021). Although this makes them highly suitable for smaller assistance tasks, their effectiveness can limit them for comprehensive medical rehabilitation programs due to limitations of force output. These assistive devices have the potential to significantly reduce the muscular effort required and improve our abilities in daily activities, representing a significant step forward in the field of rehabilitation technology.

The field of rehabilitation therapy, particularly for the elbow joint, demands innovative approaches to enhance patient outcomes. Traditional methods often rely on rigid systems, which have inherent limitations in flexibility, comfort, and customization. To address these challenges, this study proposes the design, simulation, and manufacturing of a soft pneumatic muscle specifically aimed at elbow rehabilitation. The primary objectives of this research are to investigate how different air pressures influence the movement of the soft elbow muscle during rehabilitation exercises, to analyze the impact of the geometric shape, size, and thickness of the air pillows within the soft muscle using Finite Element Analysis (FEA) to model the motion and deformation of the muscle made from thermoplastic polyurethane (TPU) material under applied positive pressures, and to fabricate the muscle using VAT photopolymerization and stereolithography (SLA) techniques, ensuring high precision and adaptability in the design. Additionally, an innovative pneumatic circuit, coupled with a LabVIEW control program, will be developed to allow precise control of the air pressure that drives the muscle’s actuation, offering enhanced customization for individual rehabilitation needs. To estimate the position of the soft elbow muscle in a 3D space, we will develop a deep learning algorithm based on the OpenCV library and YOLO V3, utilizing a real-sense depth camera for accurate data capture. By addressing these objectives, our study aims to provide a comprehensive solution for elbow rehabilitation, leveraging advancements in soft robotics and intelligent control systems to improve patient outcomes.

## 2 Soft actuators for elbow rehabilitation

**FIGURE 2:**
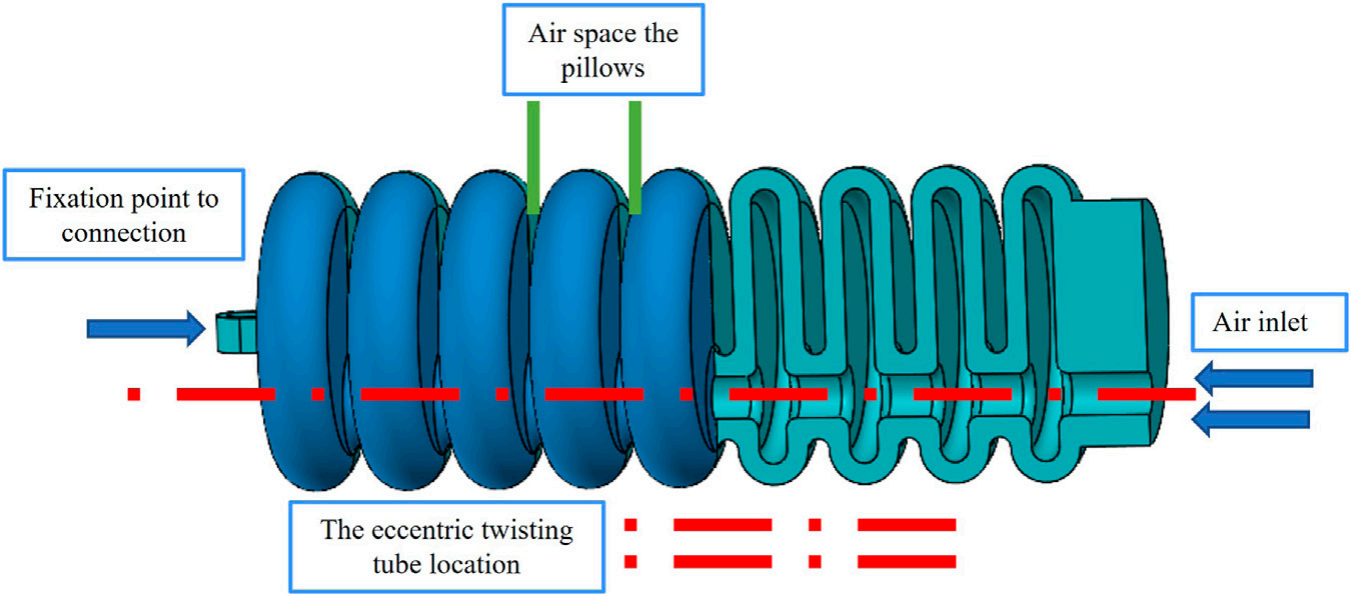
The soft artificial muscle internal details representing the internal structure and the air tubes, the affected walls with the pressure, and the rotational motion of our rehabilitation soft joint.

The soft artificial muscle proposed in the current investigation has been engineered in a manner that emulates the essential biomechanical actions of flexion and extension to enhance the patient’s functional mobility. The design of this novel muscle precisely mirrors the angular trajectory of elbow articulations, with specific geometric parameters, namely, the air cushion thickness and the intercushion separation distance, being instrumental in its function. This configuration facilitates controlled expansion, thus inducing torsion, which is crucial for the rotational dynamics of the elbow. With the introduction of external pneumatic forces, the inner lining of each air cushion is subjected to rotation and deformation, culminating in an increased rotational angle at the soft-joint interface. When all cushions are simultaneously inflated with pressurized air, the soft joint is capable of executing a complete range of motion by means of torsional mechanics. This distinctive construction is imperative for the accurate simulation of complex movements of the elbow. The integration of this advanced design into rehabilitation protocols is expected to substantially improve functional mobility of the elbow and comprehensive grasping capacity of patients. It represents a significant advance in the efficacy of rehabilitative therapy, providing improved mobility for people affected by elbow dysfunction. As delineated in Figure 2, the design formulated in this study is projected to enhance functionality and performance, serving as an assistive modality in the rehabilitation of the elbow joint.

**FIGURE 3:**
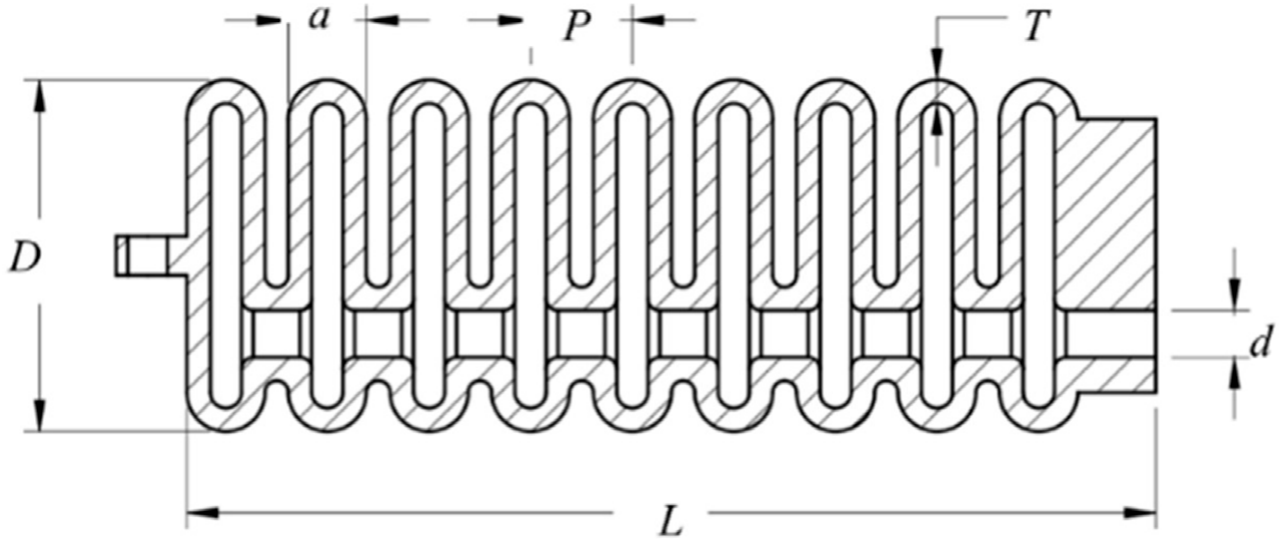
Visualization and analysis of parameters for a soft pneumatic muscle. The illustration offers a detailed representation of the soft pneumatic muscle, highlighting its design characteristics and key parameters.

In the present investigation, a rigorous methodological approach has been adopted to determine the optimal geometric parameters that are conducive to maximizing the functional efficiency of soft pneumatic actuators. A comprehensive benchmarking process was implemented, encompassing a diverse array of variables that evaluate motion range, force generation, and energy efficiency optimization. The derived model exhibits promising kinematics, which suggests potential applications in the rehabilitation of post-stroke patients and various physical therapy scenarios. An exhaustive analytical exercise was performed to acquire a complete understanding of the behavior of the soft muscle. This analysis involved evaluating muscle response under a spectrum of conditions, particularly at incremental levels of air pressure. The response of the muscle to varying pneumatic pressures was examined, providing valuable information on its performance characteristics. Figure 3 graphically presents the crucial geometric parameters that are integral to the functionality of the soft muscle. Similarly, Table 1 enumerates and explicates the detailed numerical parameters. This holistic depiction provides a concise overview of the essential design features and the underlying mechanics of the muscle. Future research endeavors and professional applications may systematically manipulate these parameters to tailor muscle performance to specific requirements. Strategic selection of geometric parameters, coupled with a comprehensive analysis performed, improves the understanding of soft actuators within the rehabilitation domain. Such advances bolster the conviction that soft muscle has significant potential to increase the efficacy of therapeutic interventions for patients recovering from strokes and those seeking restoration of motor functions.

**TABLE 1:** Detailed listing of geometric parameters defining the structural properties of the soft muscle actuators.

Parameter	Value (mm)
Length (L)	124
Outer Diameter $\left(D_{o}\right)$	45
Air inlet diameter (d)	6
Pitch (P)	13
Thickness (T)	3

The conceptual framework for the soft muscle design utilized in the present study revolves around the construction of single chamber multi-air pillows fabricated from a flexible polymer, specifically *Thermoplastic Polyurethanes* (TPUs), which are a category of polymers renowned for their ability to exhibit elastomeric characteristics. TPUs are distinguished by their versatility, which encompasses a broad spectrum of hardness levels, from highly pliable and elastomeric to firm and robust. Departing from the conventional approach of employing an elastomeric reinforcement layer, the employment of multi-air pillows, each with a singular air chamber, confers considerable structural integrity to the design. The rotational dynamics of the soft muscle was modulated through the pressurization of these air pillows. The soft muscle performance and operational behavior were characterized by critical parameters, including chamber length (L) , chamber outer diameter $\left(D_{o}\right)$ , air inlet diameter (d) , pitch (P) and chamber thickness (T) , as enumerated in Table 1. A chamber thickness of 3 mm was selected, using TPU material recognized for its expansive qualities. The judicious selection of material is acknowledged as a paramount factor that significantly impacts the soft muscle’s proficiency in executing angular rotations to a prespecified degree.

Optimization of air pressure and airflow rate is essential to achieve the desired performance of soft muscle actuators. The application of pressurized air is necessary to produce the required rotation angle within the soft muscle. Optimal muscle behavior is critically dependent on the selection of appropriate materials, as well as precision in sizing, shaping, and the regulation of both air pressure and flow rate. In the context of this study, soft muscle was found to operate effectively within a pressure range of 20–200 kPa. By meticulously considering these variables, the design and operation of the soft muscle model have been optimized to ensure consistent and precise muscle actuation. This foundational knowledge contributes to the advancement of soft pneumatic muscle development, enhancing its potential for integration into a variety of applications, including, but not limited to rehabilitation robotics and assistive devices.

## 3 FEA of TPU soft muscles

The explanation of mechanical performance in soft muscle designs is imperative for both prediction and enhancement. This level of understanding is achieved through a methodical approach that encompasses empirical testing and the deployment of sophisticated finite element simulation tools. Among these tools, Ansys software stands out due to its widespread adoption in Finite Element Analysis, as substantiated by references (Saleh et al., 2020a; Soliman et al., 2021). The soft muscle under examination is made from thermoplastic polyurethane (TPU), a material selected for its array of advantageous properties. These properties are characterized by low viscosity and superior fluidity, which collectively streamline the fabrication process. Furthermore, the inherent elasticity of TPU, its robust tensile strength, and resistance to the detriments of aging serve to significantly enhance the resilience and longevity of the functionality of the soft muscle. For a more granular exposition of these properties, Table 2 is provided as a comprehensive resource. In the realm of additive manufacturing, Thermoplastic Polyurethane (TPU) is often processed using stereolithography (SLA), which is a distinguished member of the Vat photopolymerization family. The SLA technique utilizes an intricate ultraviolet (UV) laser system that meticulously cures the polymer resin layer by layer. The resins used are in the liquid state, specifically chosen for their photosensitive thermosetting properties. Although TPU’s suitability for 3D printing is well established, given its soft and pliable nature, it also presents anisotropic properties that warrant further investigation for a full understanding of its mechanical behavior.

**TABLE 2:** Mechanical properties of thermoplastic polyurethane (TPU) for soft muscle application.

Parameter	Value
Hardness (D)	81/1
Tensile Strength (MPa)	7.56
Bending Strength (MPa)	8.76
Viscosity	497 cP
Load Deformation Temperature	59.1 °C

**FIGURE 4:**
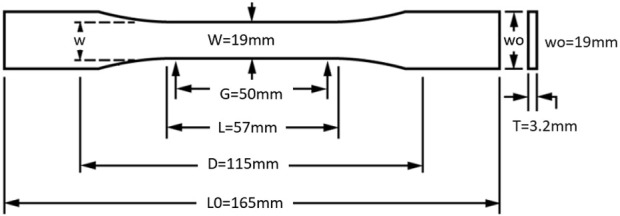
Geometrical dimensions and manufacturing of ASTM D638-14 specimens of Silicone rubber material (Dimensions in mm).

**FIGURE 5:**
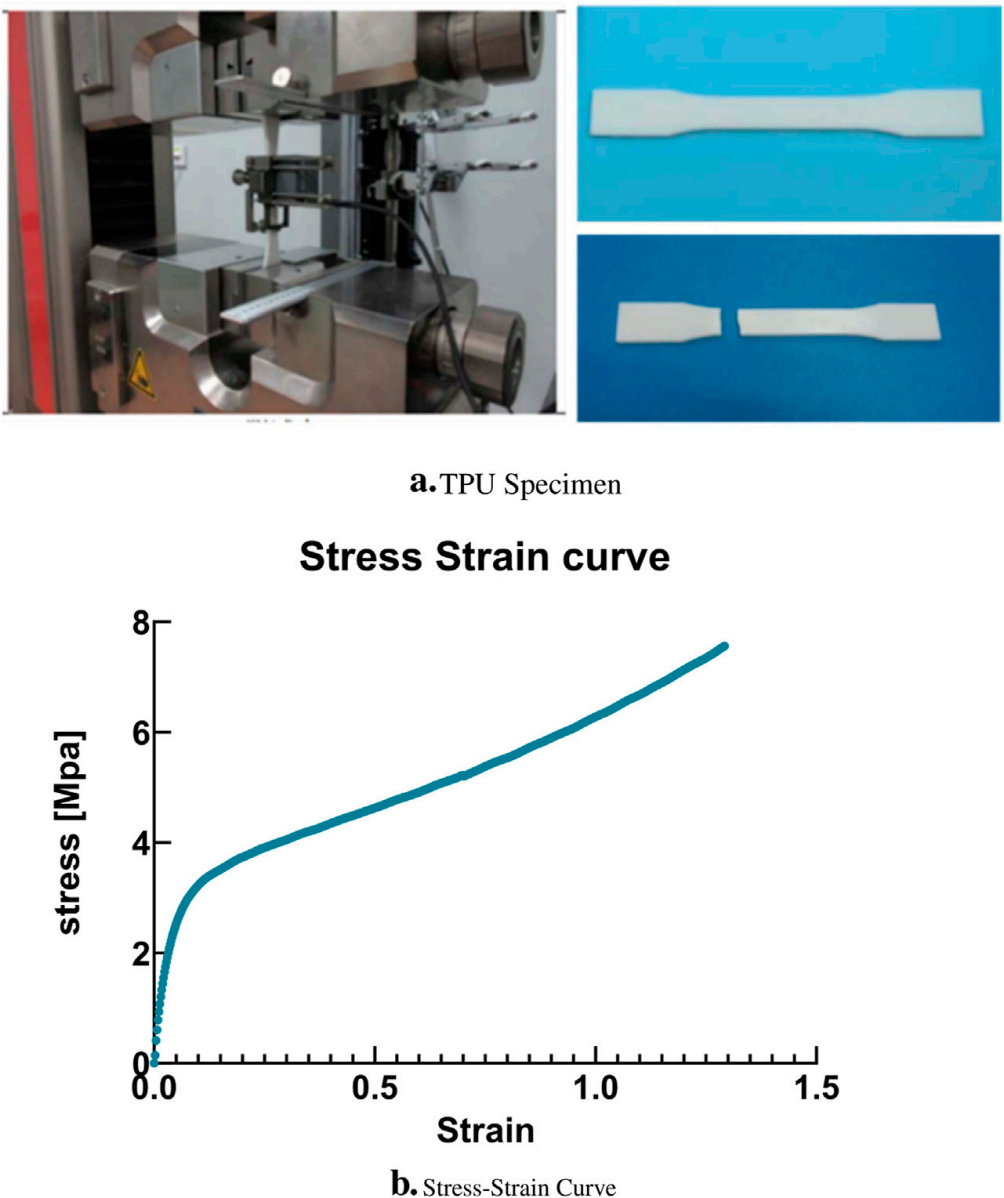
**(A)** ASTM D638-14 printed sample of TPU Specimen after curing. **(B)** Experimental results: Stress-strain curve for the TPU Specimen.

To achieve this comprehensive understanding, a series of uniaxial tensile tests have been meticulously performed on TPU samples, strictly adhering to the standards established in ASTM D638-14. These tests were performed using a universal precision testing machine. The parameters for these tests were carefully controlled, with the thickness of the sample maintained at 3.2 mm, the test speed regulated at 5 mm/min and the gauge length for the samples set at 50 mm. Figure 4 presents the actual post-fabrication TPU specimen. In addition to this, Figure 5 provides a visual representation of the test apparatus. The results of these tests are represented graphically in the stress-strain curve, as shown in Figure 5. This rigorous testing regimen is crucial to delineating the stress-bearing capacities and elastic behavior of TPU under specified conditions, contributing to the broader field of material science and engineering with empirical data and insightful analysis.

**FIGURE 6:**
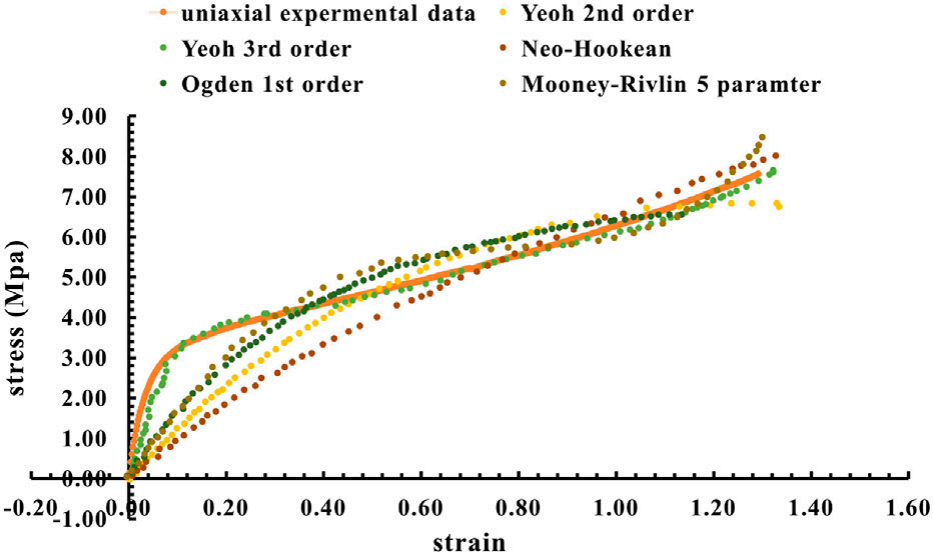
TPU 3D printed tensile specimen vs. the Yeoh third order model.

The acquisition and analysis of empirical stress-strain data is fundamental in the development of constitutive models for hyperelastic materials within the domain of Finite Element Analysis (FEA). Characterized by its non-linear and hyperelastic attributes, the thermoplastic polyurethane (TPU) material is rigorously examined under conditions that simulate the primary movements of soft muscle, longitudinal, and rotational actuation. A meticulous comparison is conducted between the experimental stress-strain curve and various hyperelastic constitutive models to ascertain the most accurate representation of the material’s behavior. Empirical evidence derived from the analysis of 3D printed TPU tensile specimens suggests a marked congruence between the experimental data and the Yeoh third-order hyperelastic model, as depicted in Figure 6. This alignment corroborates the findings of previous research, which have posited the superiority of the Yeoh model in capturing the mechanical response of hyperelastic materials compared to alternative models (Polygerinos et al., 2015b; Tawk et al., 2018). The governing equation for hyperelastic material models, which encapsulates the relationship between stress and strain in hyperelastic materials, is formalized as Equation 1 (Palmić and Slavič, 2023; Guan et al., 2020).

$$W=\overset{N}{\underset{n=1}{\sum }}\left(\frac{dk}{C_{in}}\right){\left({\bar{I}}_{1}-3\right)}^{n}$$ (1)

In the presented notation, W is defined as the strain energy function, while N signifies the total number of terms included within the summation series. The term ${\bar{I}}_{1}$ corresponds to the first invariant of the deviatoric strain, and the variable (J) denotes the determinant of the elastic deformation gradient, with the relationship ${\bar{I}}_{1}=J^{-2/3}I_{1}$ being specified. The material constants are represented by $C_{i0}$ and $d_{k}$ . In the context of the Yeoh model, the assumption of incompressibility is typically ignored. The determination of $C_{i0}$ is acknowledged as a critical step in the execution of simulations. To simplify the simulation process, the decision was made to adopt the first order approximation of the Yeoh model, indicated by setting (N) equal to 3. the simulation software requires only the provision of the material constant $C_{30}$ .

The ANSYS software suite was used for the development and execution of a finite element analysis (FEA) model, meticulously tailored to assess the mechanical behavior of the prototype under investigation. The simulation protocol involved the application of a controlled pressure range that varies systematically from 100 to 200 KPa. This range was selected to include the expected operational stresses and to evaluate the response of the material under typical usage conditions. Critical to the fidelity of the FEA model were the material parameters, which were derived from an extensive curve fitting process. This process involved a careful analysis of the experimental data to determine the precise constants that characterize the hyperelastic behavior of the material. These empirically derived parameters were then judiciously integrated into the ANSYS software, thus enhancing the accuracy of the finite element model. For a detailed exposition of the hyperelastic model parameters, one is directed to Table 3. This table has been meticulously curated to provide a comprehensive repository of the material constants, which are instrumental for both the validation of the simulation results and for facilitating a deeper understanding of the material’s behavior within the simulated environment. The availability of these parameters ensures a robust foundation for subsequent analyses, enabling researchers and engineers to predict the performance of the material with greater confidence and precision.

**TABLE 3:** The parameters of Yeoh third order model of TPU material.

Parameter	Value
TPU Density ${kg/m}^{3}$	1,137
$C_{10}$ (MPa)	3.0617
$C_{20}$ (MPa)	−0.63765
$C_{30}$ (MPa)	0.098624

## 4 FEA results discussion

**FIGURE 7:**
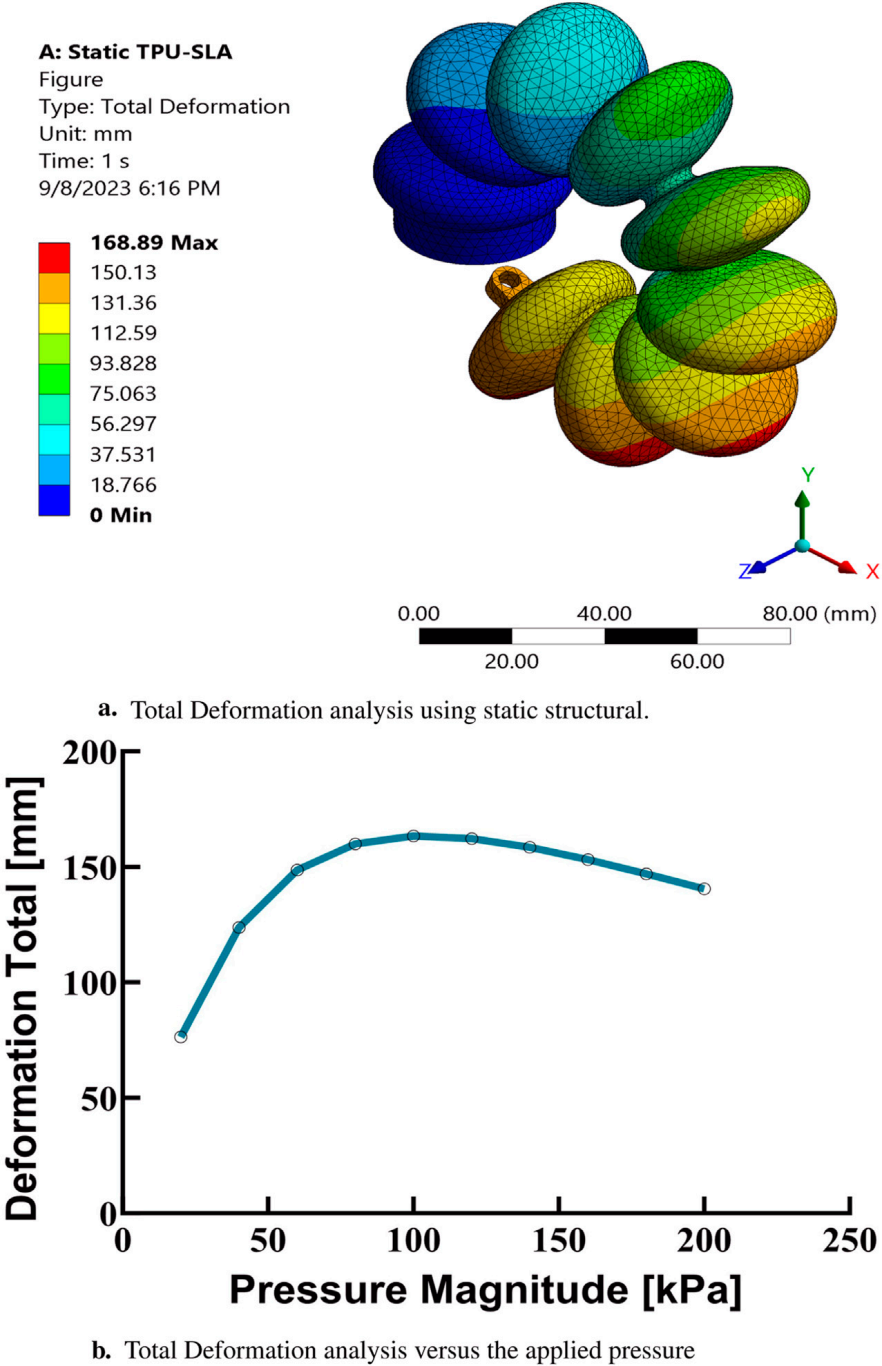
Finite element analysis results at maximum pressure based on the third-order Yeoh hyperelastic model. **(A)** Total deformation analysis using static structural simulations.**(B)** Total deformation as a function of applied pressure.

**FIGURE 8:**
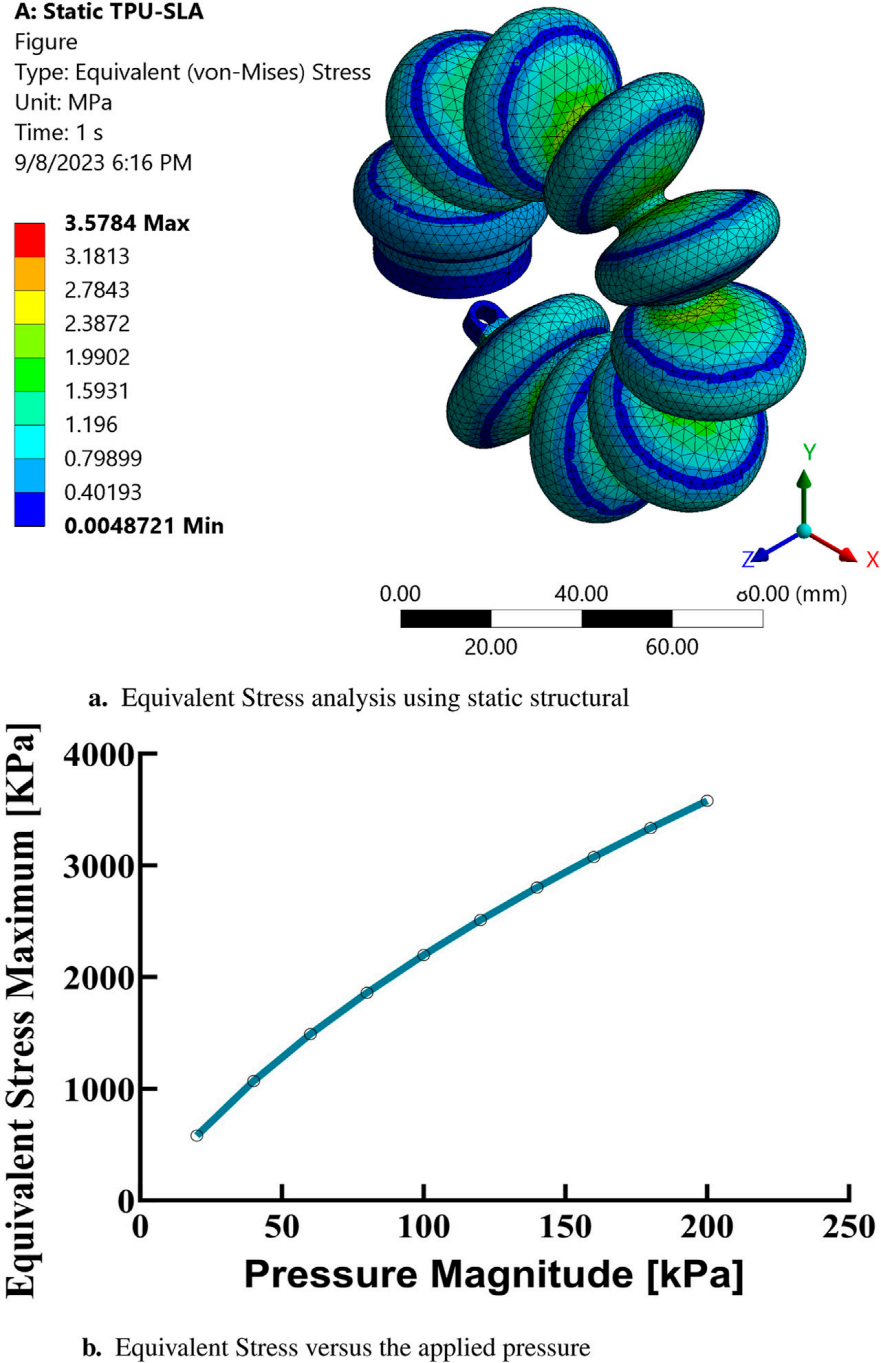
Finite element analysis of equivalent stress. **(A)** Static structural simulation results for equivalent stress at maximum pressure based on the Yeoh hyperelastic model. **(B)** Equivalent stress as a function of applied pressure.

**FIGURE 9:**
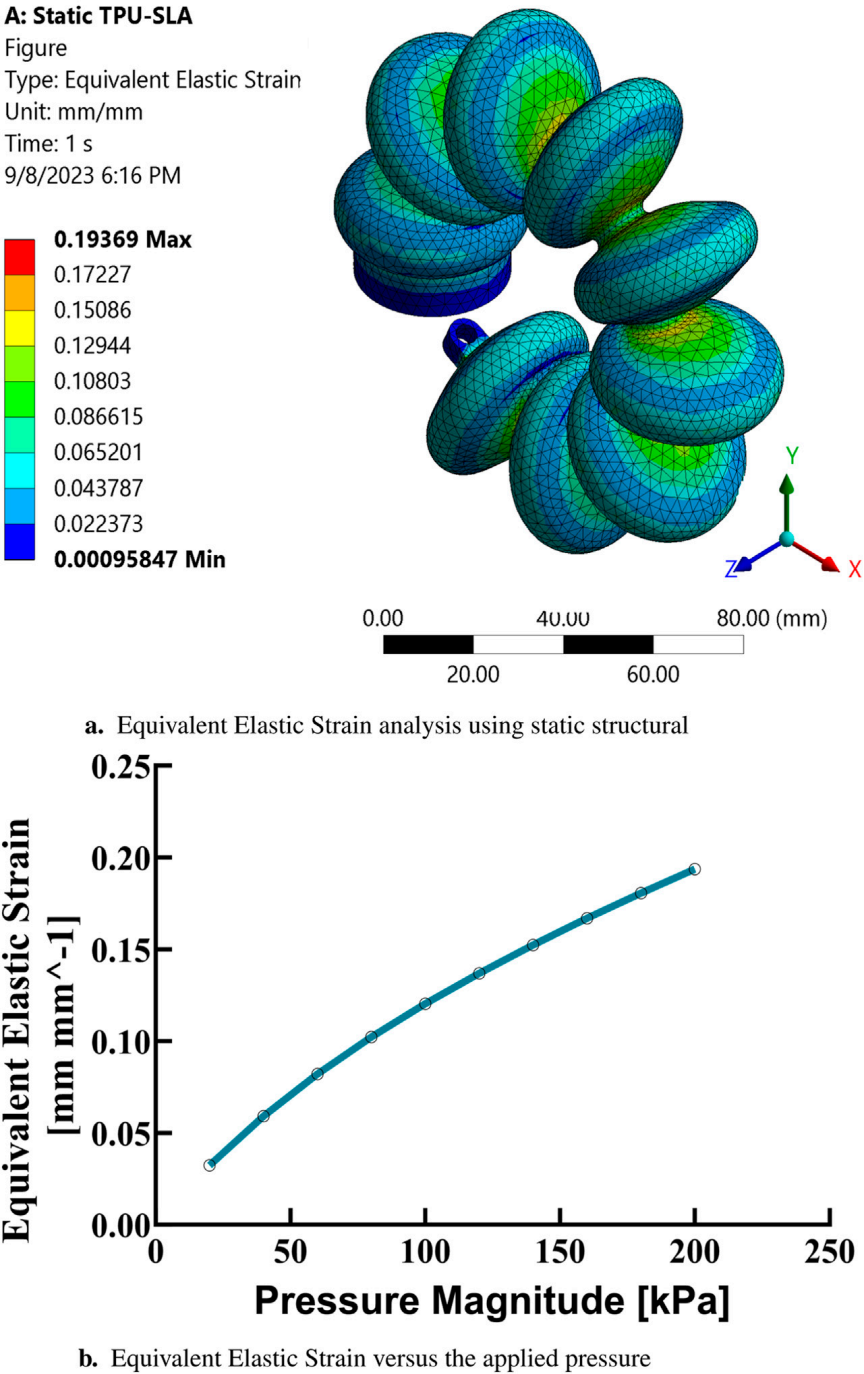
Finite element analysis of equivalent elastic strain. **(A)** Static structural simulation results for equivalent elastic strain at maximum pressure. **(B)** Equivalent elastic strain as a function of applied pressure.

In the present study, finite element analysis (FEA) has been utilized to simulate the mechanical behavior of a soft muscle, with an emphasis on its response to bending, which induces a rotational angle. The effect of varying the positive pressure of the inlet on this response has been systematically investigated. Ansys Multi-Physics 2021 R2TM software has been used for these FEA simulations. The simulations covered a range of input pressure conditions, from 100 to 200 kPa, in increments of 10 kPa. The analyzes were conducted in three dimensions and incorporated non-linear mechanical properties to facilitate a large deflection analysis. Quadratic order tetrahedral elements were selected for mesh generation, with an element size of 3 mm. This size was determined to be optimal after a comprehensive evaluation of the granularity of the mesh, examining sizes from 1 to 6 mm to ensure convergence. The purpose of these simulations was to evaluate the mechanical response and performance of a soft pneumatic muscle, as used in elbow rehabilitation devices, under various loading conditions. The findings of this extensive simulation analysis are graphically depicted in Figures 7A, B, 8A, B, 9A, B. These results contribute significantly to understanding the complex mechanical behavior of the soft elbow muscles and establish a foundation for future developments and optimizations in soft robotics, with a particular focus on applications in elbow rehabilitation. A meticulous examination of the influence of different input pressures was conducted, with the objective of acquiring in-depth knowledge of the deformation behavior and stress responses of the soft pneumatic muscles.

**FIGURE 10:**
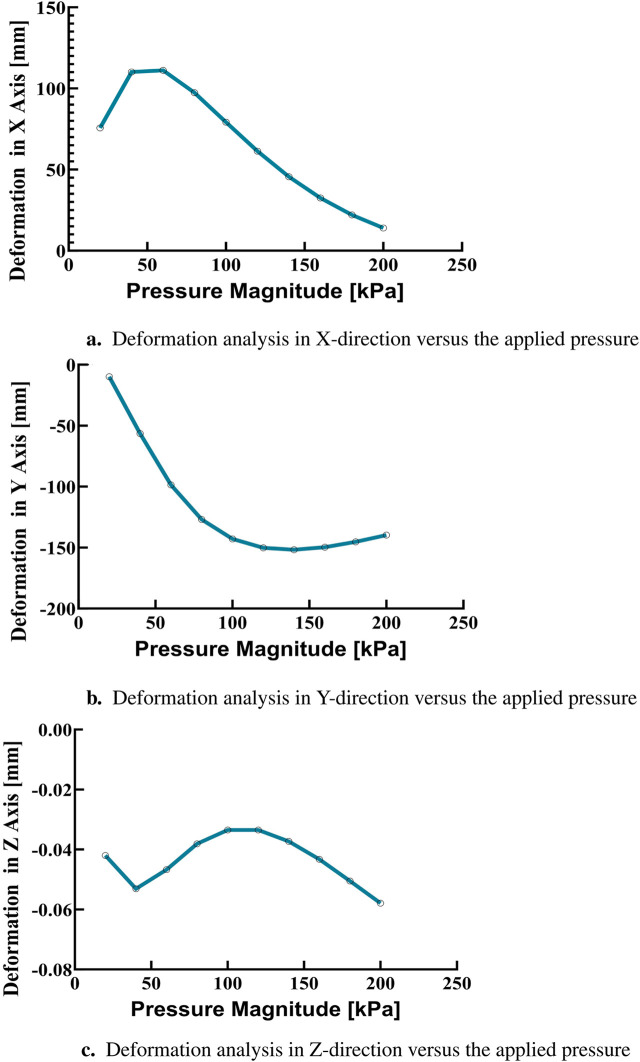
Finite element analysis of deformation in the X, Y, and Z directions. **(A)** Deformation in the X-direction as a function of applied pressure. **(B)** Deformation in the Y-direction as a function of applied pressure. **(C)** Deformation in the Z-direction as a function of applied pressure.

**FIGURE 11:**
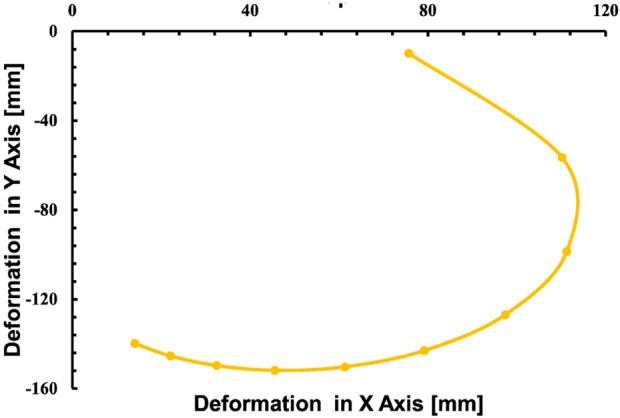
Relationship between the deformation in the X-direction with respect to the Y-direction.

In the simulations conducted, a consistent observation was made across all pressure points: positive deformation was noted along the X-axis and negative deformation along the Y-axis, with deformation magnitudes ranging approximately from 10 to 150 mm, displaying a nearly equal distribution at all examined points. These findings indicate that the muscle manifests a circularly symmetric rotational behavior within the XY plane. In contrast, deformations along the Z-axis were found to be negligible, with a maximum measured value of 0.003 mm, suggesting an insignificant degree of elongation as presented in Figures 10A–C. This result confirms the absence of disturbances in control parameters and confirms the stability of the elbow muscle during its actuation within the XY plane as presented in Figure 11. These observations emphasize the specific response of the soft muscle to the action of pressure along the intended XY direction, underscoring the stability of its movement. Furthermore, muscle controllability has been shown to be pressure-dependent, allowing a range of rotational motion from 0 to 360°. This range of motion is instrumental in controlling various angles of the elbow during rehabilitation exercises. The adaptability and controllability observed in muscle behavior are indicative of its ability to regulate the rotational angle of the elbow with precision. This characteristic is particularly beneficial for tailoring rehabilitation protocols to the individual needs of patients, thus improving the customization and effectiveness of the rehabilitation process.

## 5 Soft muscle fabrication

**FIGURE 12:**
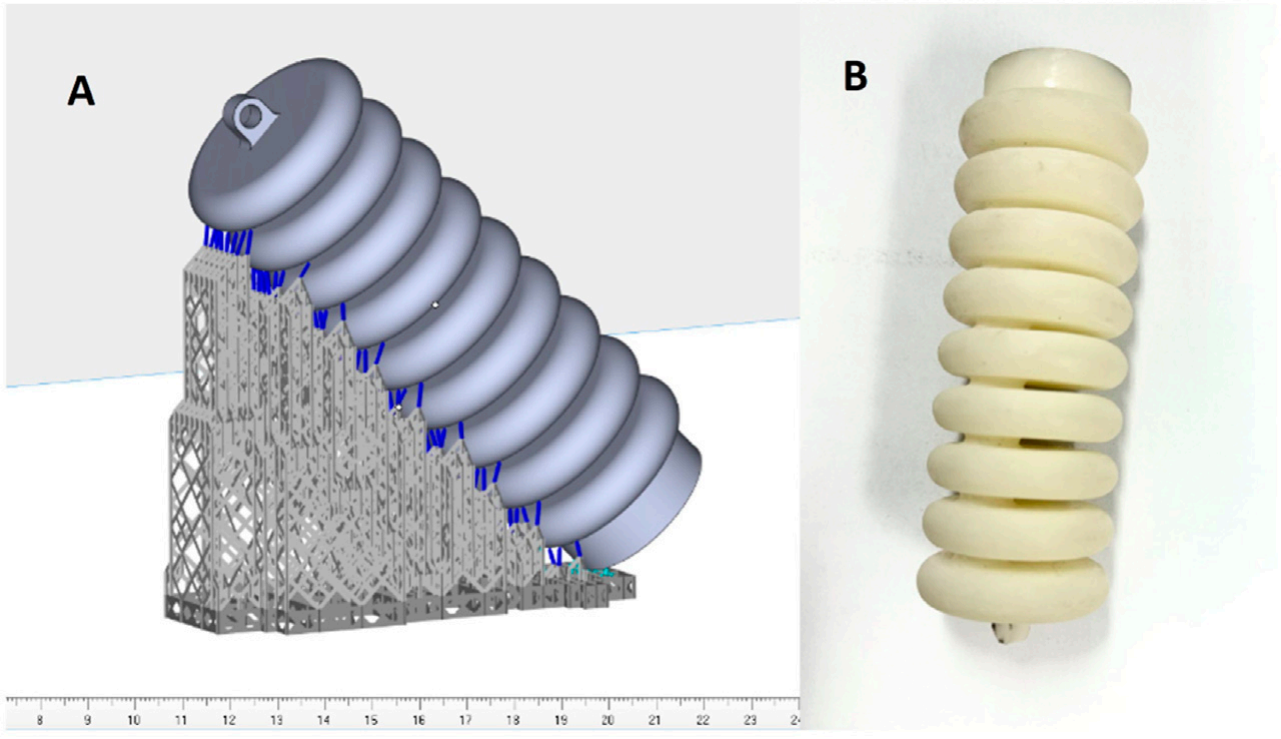
**(A)** Demonstrates the 3D printing software view and. **(B)** Demonstrates the final soft muscle printed using TPU material with the SLA method.

The field of soft muscle fabrication has undergone considerable innovation thanks to recent studies that have championed a host of new manufacturing tactics. Among these, the integration of additive manufacturing technologies has been a game-changer, with 3D printing methods like Selective Laser sintering (SLS), Fused Deposition Modeling (FDM), and Stereolithography (SLA) spearheading the development of intricate soft pneumatic actuator designs. Customization is particularly crucial in the production of rehabilitation devices to meet the specific ergonomic needs of individual patients, prompting the shift toward flexible manufacturing techniques that can adapt to a diverse array of patient sizes. The stereolithography (SLA) process, which is a member of the Vat photopolymerization additive manufacturing category, has traditionally been employed to craft soft muscles. This method intricately cures a layer of photosensitive thermoset polymer resin using an ultraviolet (UV) laser according to the computer-generated design of the soft muscle as shown in Figure 12. SLA has a distinct advantage over traditional molding by mitigating risks such as air leakage that can negatively influence muscle functionality and movement paths. It also excels in making muscle as a single entity, eliminating the assembly of multiple pieces to form the end product. In this context, Thermoplastic Polyurethane (TPU), with its unique ability to solidify from liquids under the layer-by-layer application of SLA, stands out. TPU maintains the necessary mechanical properties for the desired shape and is ideal for applications requiring both flexibility for twisting and durability to withstand high pressures, as is the case with elbow motion during rehabilitation exercises.

In this study, the SLA technique was used to create a soft actuator from TPU. The operation began with the detailed segmentation of the computer-assisted design (CAD) model into successive layers, using a slicing program to generate the required g code for 3D printing. The actuator took shape with the help of the Luen Tai Light Curing 3D Printer, backed by Taizhou Shenggu 3D Technology Co., Ltd., and the design refinement was handled by Materialize Magics 21.0 software. We meticulously set the printing parameters: a layer height of 0.1 mm, a laser power of 800, and an infill density at 100%. Laser power proved to be a critical component in the printing process; too high a setting resulted in parts that were too stiff, hindering the necessary flexibility, while too low a setting weakened the parts, affecting their load-bearing capacity for elbow elevation in rehabilitation exercises. After a series of experiments, we found the optimal laser power to be 800, hitting the sweet spot for producing a soft muscle actuator that met all the structural and functional needs. These conclusions are drawn from a detailed experiment anchored in the data specifically collected for this research.

## 6 Elbow rehab actuator test rig

**FIGURE 13:**
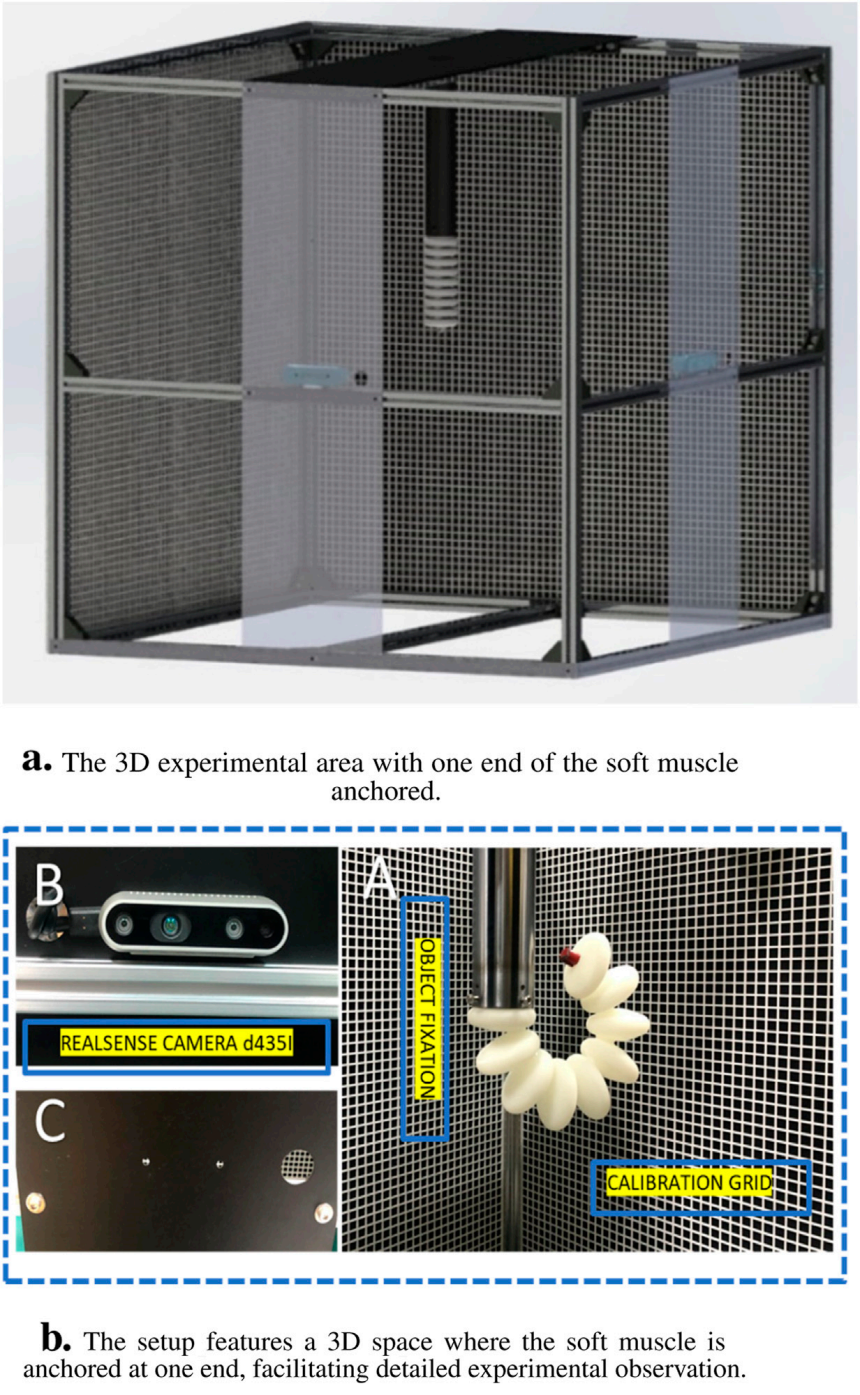
The experiment platform for measuring the elongation in X-axis regarding the pressure head change. **(A)** The experiment space where the soft muscle fixed from one end in 3D space, **(B)** the reaLsense D435i camera detecting a video for the movement, and **(C)** the laser cut plate for camera fixation.

An experimental apparatus was meticulously designed and constructed to facilitate the study of soft muscle deformation under actuation at varying pressures, specifically within the range of 100–200 kPa (KPa), for applications in elbow rehabilitation. The apparatus was composed of four principal components: a three-dimensional (3D) environment to allow unrestricted movement and deformation of the soft muscle, a pneumatic circuit for pressure delivery, a control system using LabVIEW software, and a deep learning algorithm employed for position estimation within the 3D space using an Intel RealSense D435i sensor. In the initial setup phase, the soft muscle was securely anchored at one end within a cubic cell, measuring 1 m × 1 m, designated as the workspace. This cell featured two laser cut grid plates placed on intersecting walls behind the muscle to serve as reference structures, thus ensuring precise displacement measurements throughout the experimental trials, as illustrated in Figure 13. The grid, strategically placed at a fixed distance from the muscle, was delineated in contrasting colors to facilitate visual differentiation of muscle displacement. This spatial arrangement was critical for the accurate observation of muscle behavior when subjected to differential pressure scenarios. Furthermore, to provide the pressurized air necessary for actuation and to ensure the stability of the soft muscle, a cantilever support system was implemented. This support was attached directly to the muscle and extended from the upper boundary of the cubic cell, allowing the maintenance of a consistent actuation environment.

**FIGURE 14:**
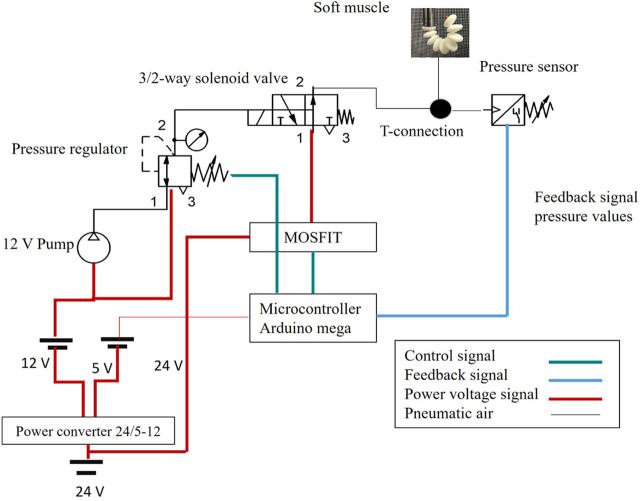
Schematic drawing for the pneumatic circuit and the electrical drivers to control the soft muscle.

The pneumatic circuit of the experimental platform was a cornerstone in the precise regulation of pressure levels and flow rates directed at the soft muscle as shown in Figure 14. Bifurcation of the system into hardware and software components allowed granular control over muscle response to a variety of pressures, significantly improving the precision and adaptability of experimental procedures. On the hardware front, the pneumatic system was an assembly of meticulously selected electronic components. The primary power source, which provided a stable 24V supply, was the lifeblood of the pneumatic circuit. To meet the circuit’s diverse voltage needs, including 24V for the main supply, 12V for intermediate components, and 5V for logic circuits, a versatile buck converter, a 25V 2A step-up/down voltage regulator, was deployed. This regulator was adept at transforming the single input voltage into multiple required output voltages, as depicted in Section (A) of the schematic.

**FIGURE 15:**
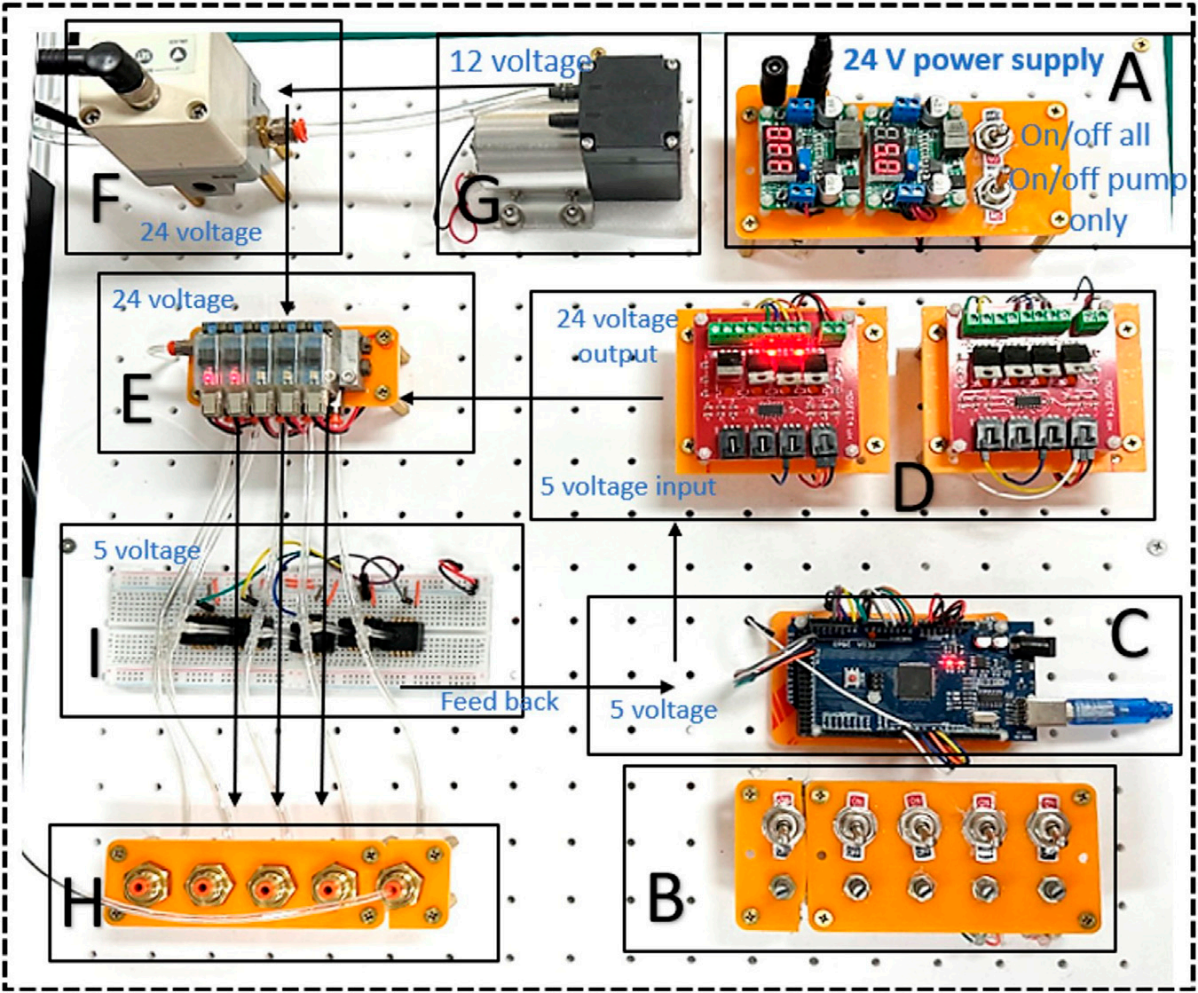
Presents the pneumatic control board, consisting of **(A)** Electric power regulator 5V AND 12 V, **(B)** 5 Potentiometer and 5 Toggle switches, **(C)** Arduino MEGA, **(D)** Power MOSFETs 24V, **(E)** 5 SMC values V1145M, **(H)** Air outlet, **(I)** Pressure sensor.

The converter was adeptly configured to seamlessly drop the voltage to 12V and 5V to meet the demands of various circuit components. An additional layer of operational security was provided by the integration of two strategically placed power switches. The first served as the master switch for the entire electronic setup as shown in Figure 15. At the same time, the second was specifically assigned to the air pump, located at position (G) of the diagram. Selective activation of the air pump was a crucial design element, as premature operation could cause a host of problems such as overheating, reduced efficiency, motor damage, and excessive noise and vibration, not to mention the potential for accelerated wear and an undue increase in internal pressure that could compromise system integrity. Position (F) of the layout was occupied by a precision pressure regulator, a component that is tasked with receiving air from the air pump and regulating its pressure before delivery. The air pump, by design, did not exert control over the output pressure, placing the onus on the regulator to ensure a stable and precisely controlled pressure output. The regulated air was then channeled to a manifold located in (E), which served as the central hub for air distribution to five pneumatic valves. Each valve was calibrated to deliver specific pressure to the soft muscle, ensuring a highly controlled actuation environment.

The software control system was anchored by an Arduino board, featured in (C), which was powered by a 5V output from the step down voltage regulator. Arduino’s role as the central processing unit of the circuit was multifaceted. It was primarily responsible for the management of three critical components of the system. The power MOSFETs in (D) were under Arduino’s command and tasked with actuating the pneumatic valves through precise opening and closing sequences. These MOSFETs were capable of modulating pressure through pulse width modulation (PWM), offering a sophisticated means of fine-tuning the pressure delivered to the soft muscle. The dual-sided nature of the MOSFETs facilitated the interface between the Arduino 5V control signal and the 24V valve operation. An alternate control mechanism was available through the regulator in (F), providing a unified pressure adjustment option for all valves simultaneously. The second key function of the Arduino involved adjusting the pressure regulator’s output through its PWM pins. The final and most important role of Arduino was to collect feedback from the integrated pressure sensors. This feedback was critical for monitoring the circuit’s pressure dynamics and ensuring that the system was airtight and functioning as intended. The intricate design and integration of the pneumatic system’s hardware and software forged a high-fidelity experimental setup. This system was instrumental in offering a nuanced simulation environment to scrutinize the dynamic behavior of soft muscle actuators across a spectrum of pressures. The ability to simulate and analyze these conditions with such a high degree of control and precision provided an invaluable tool in the study of soft muscle mechanics.

**FIGURE 16:**
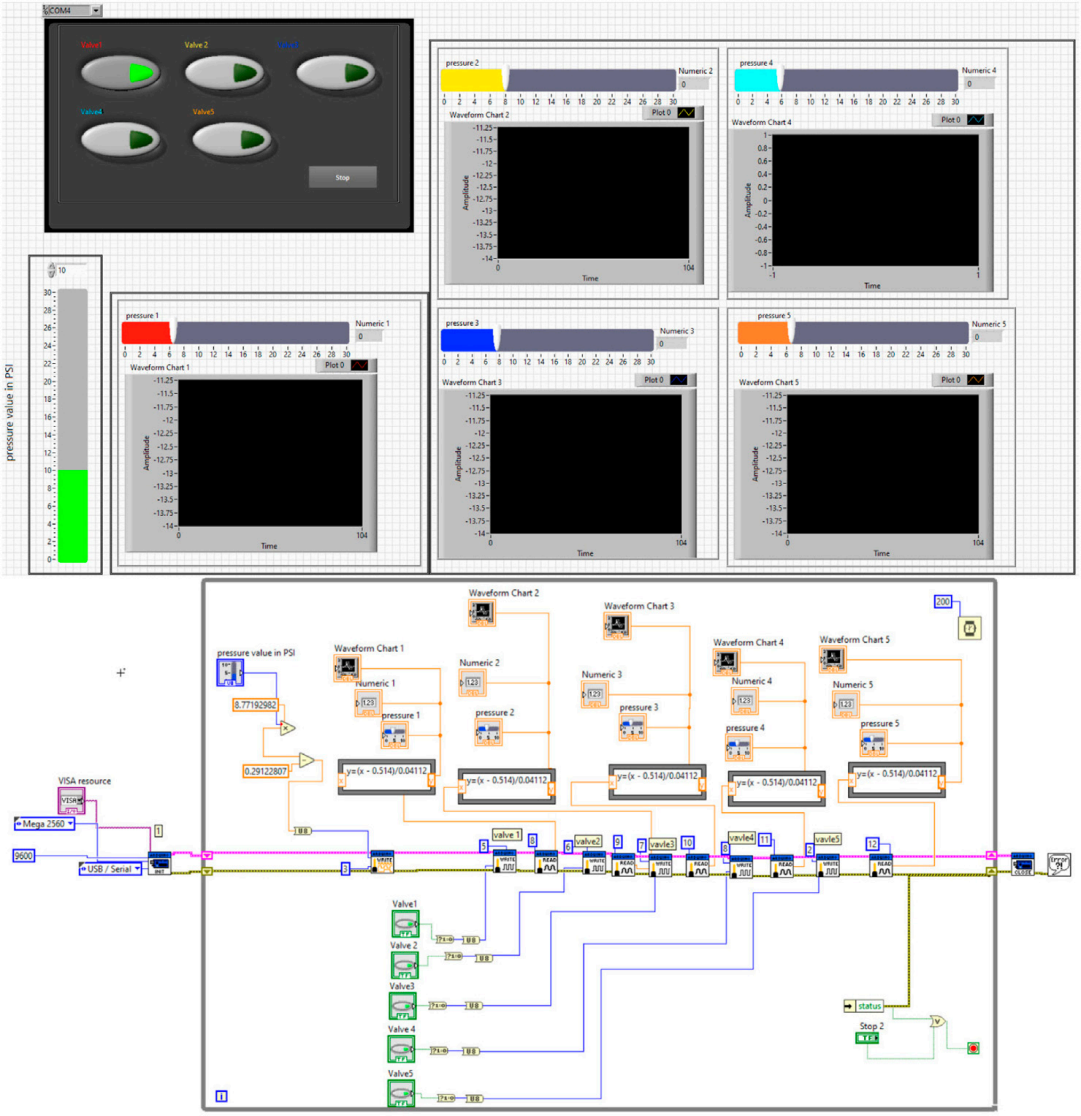
LABVIEW interface with the pressure sensor and the air pump to control the soft muscle actuation.

Third, the development of the control program was systematically carried out using LabVIEW software, based on the pre-established design of the pneumatic circuit as shown in Figure 16. The calibration phase involved meticulous adjustments to the pressure sensor and the pump, using a precalibrated pressure regulator with PSI readings. This essential step ensured the accuracy of the system’s measurements, leading to the determination of the calibration equations, which were mathematically expressed as Equations 2, 3.

$$y=\frac{x-0.514}{0.04112}$$ (2)

y=8.77192⋅x−0.29122807 (3)

These Equations 2, 3 represent the linear relationships between the sensor readings (x) and the actual pressure values (y), which serve as critical components for the system feedback loop. Subsequent to the calibration process, the mathematical model was solidified and visualized. The control software, designed to be interactive, accommodates touch-screen functionality, thereby enhancing user engagement. Specifically, the software provides two modes of pressure input: the first is a touch-based interface that allows users to set desired pressure levels with simple finger gestures; the second is a numerical input method designed to enter precise pressure values, especially when dealing with small decimal numbers. The interface’s design also incorporates five virtual buttons, each dedicated to the operation of a specific pneumatic valve. This feature simplifies the process of managing the pneumatic system.

In addition, the software includes five distinct screens, each corresponding to a particular segment of the pneumatic circuit, to continuously display real-time pressure data. The ergonomics of the design were carefully considered to ensure that the user interface, shown in the upper part of Figure 16, is intuitive and user-friendly, while the developer interface, located in the lower part of the figure, provides comprehensive control and monitoring capabilities for advanced users or system technicians.

Fourth, the experimental protocol was developed with precision to accurately document the complex dynamics of muscle movement, a critical component for the analytic study of muscle behavior under various stimuli. A high-resolution RealSense D435i camera was strategically positioned to capture high-fidelity video at a rate of 30 frames per second, ensuring detailed visualization of the muscle’s trajectory in three-dimensional space. This camera, selected for its superior resolution and depth-sensing capabilities, facilitated an exhaustive record of the muscle’s morphological changes in response to differential pressure conditions. The recorded video data was not only high in resolution but also meticulously synchronized with the pressure readings and other relevant experimental parameters, utilizing the capabilities of the LabVIEW software. This integration allowed for a comprehensive time-stamped dataset, which captures the simultaneous interplay of visual and sensor-based data, which is indispensable for subsequent detailed analysis and modeling of muscle behavior.

## 7 Rotational (bending )angle experiments

**FIGURE 17:**
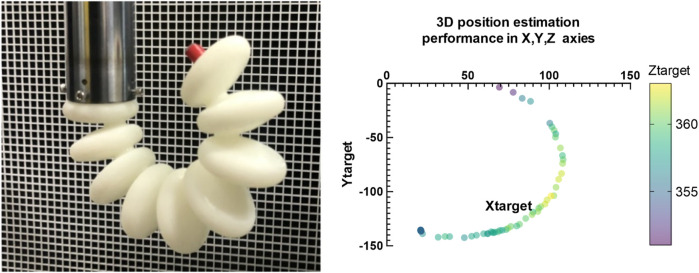
Shows the real performance on the elbow muscle during the pressure actuation and the shape during the experiment over time which is identical to the elbow muscle performance.

**FIGURE 18:**
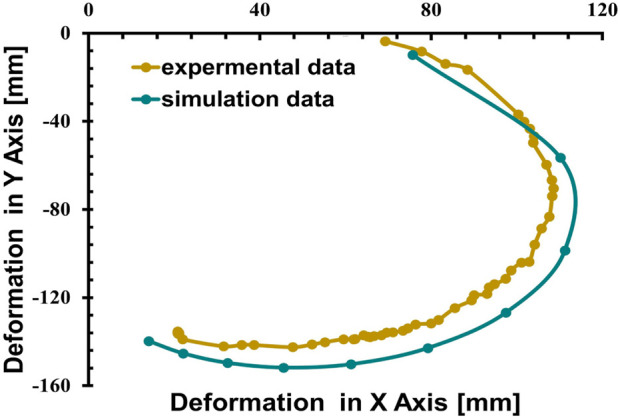
Demonstrates the correlation between the deformation in Y-axis with respect to the change of the pressure in the Simulation compared to experimental tests.

The camera was strategically positioned at a predetermined distance from the muscle, with an orientation perpendicular to the grid sheet to facilitate accurate three-dimensional displacement measurements of the muscle. To elucidate the relationship between head pressure variations and the rotational angle of the soft muscles, which is critical for elbow rehabilitation applications, deep learning models were used for position estimation. These models were able to accurately determine the three-dimensional coordinates (x, y, z) of the muscle’s end tip, which was distinctly marked with a red indicator. The models utilized OpenCV and YOLOv3, the latter being an end-to-end neural network that simultaneously predicts bounding boxes and class probabilities, contrasting with previous object detection algorithms that adapted classifiers for detection tasks. The algorithm calculated displacement relative to a calibrated initial position of the muscle’s end tip, established through a calibration procedure prior to the measurement phase. Subsequent changes in position were graphically depicted in relation to pressure alterations. Figures 17, 18 provides a visual comparison between the initial rotational angle of the muscle and its subsequent transformation under varying pressure conditions during actual experimentation, alongside the simulated results. There was a notable correspondence between the simulated and experimental data, indicating the precision and reliability of both the experimental apparatus and the position estimation algorithms.

To quantify changes in the coordinates of the muscle and finger during actuation at different pressures, an algorithm was developed to predict movement based on input pressure. This algorithm utilizes a depth camera to determine the Z-axis values, from which the X-axis values are inferred. Initially, a perspective projection transformation is applied; the first step involves assuming the camera’s center coincides with the world axis origin and the image plane is positioned in front of the projection origin. The coordinates of a point on the image plane are calculated using triangulation based on similar triangles. Subsequently, the following steps are taken.

1. Conversion of camera coordinates to image coordinates is performed.
2. The Equation 4 define the coordinates X and Y :

$$x=z\cdot \frac{\left(u-c_{x}\right)}{f_{x}},\;y=z\cdot \frac{\left(v-c_{y}\right)}{f_{y}}$$ (4)

The algorithm, derived from a foundational theoretical framework, exhibited a formidable predictive accuracy of 99.8%. For training purposes, an extensive dataset comprising 250 meticulously captured images of muscles and fingers was used, which provided a robust basis for the algorithm’s learning process. After completion of the training regimen, the algorithm was subjected to a rigorous experimental evaluation to determine its effectiveness in identifying positional changes within muscle tissue during active movement. This evaluation involved the deployment of a sophisticated camera system, configured to capture a high-resolution stream of images at a rate of 30 frames per second. This capability allowed for a comprehensive capture of dynamic sequences, effectively yielding 30 distinct images of the subject per second, each of which was quickly processed by the algorithm to determine precise measurement parameters. The output generated by the algorithm, specifically the coordinates delineating the trajectory of the object throughout the experimental procedure, was meticulously documented within an Excel file for subsequent analysis. The recorded results, as shown in Figures 17, 18 serve as a testament to the robust performance of the algorithmFig. Ultimately, using this advanced algorithmic approach not only enables the derivation of a quantitative relationship between applied pressure and the corresponding changes in spatial coordination (x, y, z), but also enhances the predictive modeling of movement for an engineered elbow muscle.

Algorithm 1 The Pseudo Code for the developed position estimation algorithm for object tracking using YOLO library and depth camera.

** Input:**
** Output:**
1 **Procedure** YOLO *Class Initialization*
2 Initialize default settings;
3 Load YOLO model, anchors, and classes;
4 Generate colors for bounding boxes;
5 **Procedure** detect_image(*image*)
6 Initialize timer, preprocessing, and YOLO detection;
7 Calculate $X_{target},Y_{target},Z_{target}$ from. $X_{temp},Y_{temp},Z_{temp}$ ;
8 Draw bounding boxes, record coordinates, calculate FPS;
9 Display output frame and check for ‘q’ key to exit;
10 **Procedure** close_session
11 Close the YOLO session;
12 **Procedure** detect_video(*yolo*)
13 Initialize camera, RealSense config, and CSV writer;
14 **while** *true* **do**
 Process and detect objects in frames using detect_image(*image*);
 Calculate coordinates, draw bounding boxes, write to CSV;
 Display color frame, FPS, and check for ‘q’ key to exit;
15 **end**
16 **Main Program:**;
17 yolo ← Create instance of YOLO class;
18 detect_video(*yolo*);

## 8 Force experiment

**FIGURE 19:**
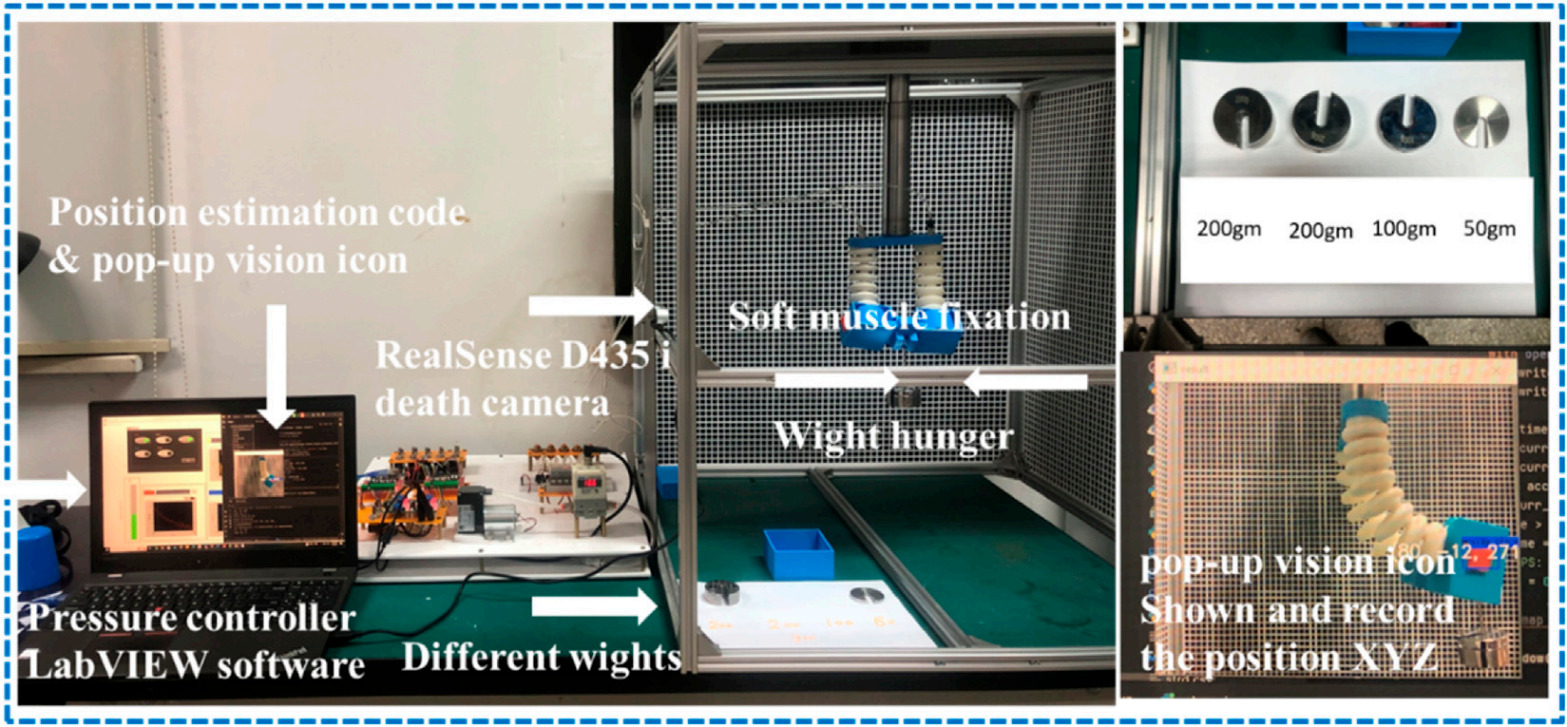
Lifting different wight experiment setup.

The experimental setup for measuring the effect of the different wights on the muscle performance in XY plane also examines the muscle maximum carrying wight is depicted in Figure 19. The soft muscle, designed as per the proposed model, is mounted vertically in a 3D printed housing to mimic the elbow designed scenario also to hang the different wight during the experiment, a set of different wights 200 gm,100 gm,50 gm, and 20 gm which is the metallic hunger wight were used to measure the maximum wight that the muscle can carry to verify if the muscle can carry the human elbow wight or not. The response of the soft muscle was evaluated under a different level of wight (170,220, 320, 370, 470) gm, and different ranging of pressure from 1 to 200 kPa with step 3.3 kPa.

**FIGURE 20:**
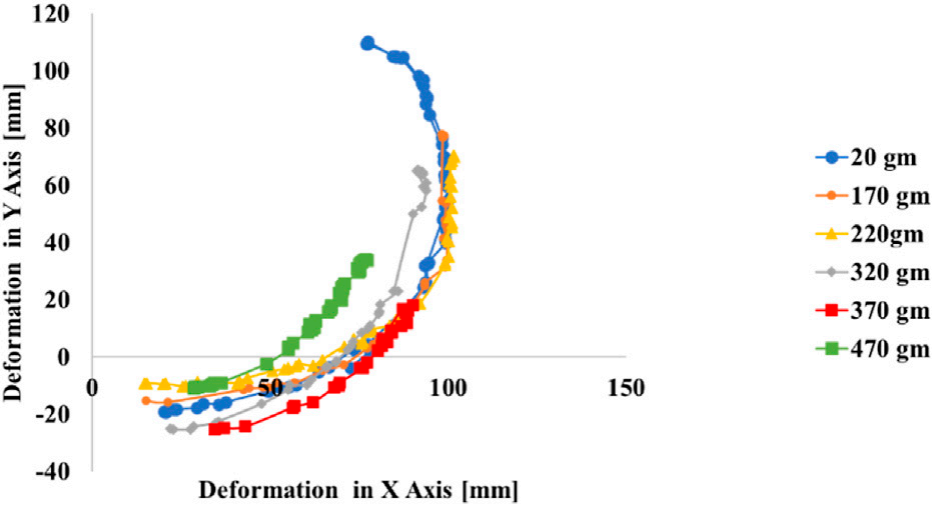
Effect of different wight on the muscle performance in XY axes.

Figure 20 Presents the performance of the soft muscle with input pressure increase the muscle angular movement increase within XY plan and. It can be observed that there is a direct relation between the pressure and the position in XY plan, and an inverse relationship between the wight increase and position in XY plan. Figure 20 provides the progression of the actuator twisting in XY plan. The 470 gm exhibited the lowest twisting in XY plan while the 170 gm was the highest twisting in XY plan which highlights the inverse relationship between the wight and the twisting angle. The resultant twisting profile form the experiment can be attributed as evidence that the bending soft muscle can lift a human elbow with 470 gm during the rehabilitation process. Using the relation between pressure, the elbow wight and the bending profile from the position estimation algorithm the rehabilitation program can be applied with high control on the elbow rotational angle without going in bending angle complicated calculations.

## 9 Conclusion

The development and comprehensive evaluation of a soft pneumatic muscle within this study have been crucial to the advancement of elbow rehabilitation techniques. The muscle was intricately designed to replicate the complex biomechanics of elbow articulation, specifically focusing on the rotational movements that are imperative for grasping the wrist and enhancing the elbow’s range of motion. Through meticulous engineering and geometric optimization, the soft muscle achieved a high degree of precision in rotational control, with its performance rigorously quantified under various conditions. Finite element analysis, aligned with exhaustive experimental testing, substantiated flexibility, precise response to control inputs, and functional reliability of the soft muscle. In particular, at an operational pressure of 200 kPa, the muscle demonstrated significant positive deformation along the X-axis and negative deformation along the Y-axis, with consistent displacements observed between 10 and 150 mm. This uniformity in deformation accentuates the muscle’s capability to exhibit rotational behavior in a circular symmetric pattern within the XY plane, which is crucial for mimicking natural joint movement. In contrast, the Z-axis deformations were minimal, peaking at a mere 0.003 mm, thereby confirming the actuator’s minimal elongation and reinforcing its stability during operation. Bending soft muscle can also lift a human elbow with 470 gm during the rehabilitation process. Using the relation between pressure, the elbow wight and the bending profile from the position estimation algorithm the rehabilitation program can be applied with high control on the elbow rotational angle without going in bending angle complicated calculations. The absence of fluctuations in the control parameters further validated the precision of the soft muscle’s movements, highlighting its fidelity to the intended rotational plane. Furthermore, muscle performance across a spectrum of pressure levels was characterized by an impressive degree of controllability, with the ability to command rotational angles ranging from 0 to a full 360°. This level of adaptability and precision allows one to fine-tune the rotational angle of the elbow, which is vital for patient-specific rehabilitation protocols. The bespoke experimental apparatus comprising a finely tuned pneumatic circuit, robust controller, immersive 3D testing environment, and an advanced position estimation algorithm emerged as a reliable and accurate platform for exploring the soft muscle’s kinematics. The experimental results, coupled with the deep learning algorithm, provided high-precision motion tracking of the soft muscle under variable pressure conditions, ensuring detailed documentation of its response spectrum. These achievements mark a significant contribution to the field of soft robotics, especially within the realm of medical rehabilitation. The results of this research not only underscore the potential of soft pneumatic muscles in therapeutic applicationsbut also lay a solid foundation for future innovation and exploration in this domain. The insights gleaned from this study are poised to inform future advances and underscore the possibility of customized patient-centered rehabilitation strategies that harness the potential of soft robotic actuators.

## Data Availability

The raw data supporting the conclusions of this article will be made available by the corresponding author, upon reasonable request.
